# Do Syrian refugees have increased risk for worser pregnancy outcomes? Results of a tertiary center in İstanbul

**DOI:** 10.4274/tjod.64022

**Published:** 2018-03-29

**Authors:** Emre Sinan Güngör, Olcay Seval, Gülşah İlhan, Fatma Ferda Verit

**Affiliations:** 1 Süleymaniye Maternity Research and Training Hospital, Clinic of Obstetrics and Gynecology, İstanbul, Turkey

**Keywords:** Syrian refugees, cesarean delivery, obstetric outcome, maternal complication, perinatal outcome

## Abstract

**Objective:**

To compare obstetric and perinatal outcomes of Syrian refugee pregnants and Turkish counterparts who gave birth at a tertiary center in İstanbul.

**Materials and Methods:**

A retrospective study including the birth records of 704 Syrian refugees and 744 Turkish pregnant women between January 2016 and May 2017 were analyzed. Demographic data, obstetric and neonatal outcomes were compared. The primary aims of this study were to evaluate the pregnancy outcomes and cesarean rates between the groups. The secondary outcomes were the use of antenatal vitamin supplementation, hemoglobin-hematocrit values, and maternal complications.

**Results:**

Our results showed that the use of folic acid and iron supplementation rates during pregnancy were similar between the groups (folic acid supplementation 8.1% vs 6.5%, p=0.264; iron supplementation 20.7% vs 19.6%, p=0.125; respectively for Turkish women and Syrian refugees). Cesarean rates were significantly higher for Turkish patients than in Syrian refugees (42.7% vs 32.7%; p<0.05). Gestational age at delivery was significantly higher among Turkish women when compared with Syrian refugees (37.7±2.3 vs 36.4±2.3 weeks, p<0.05), but there was no significant difference regarding the birtweights’ of the newborns (3134 g vs 3066 g for Turkish women and Syrian refugees, respectively, p=0.105). Although obstetric complications were seen more often in Syrian refugees, it did not reach statistical difference (9.7% vs 8.1%, respectively, p=0.285).

**Conclusion:**

Syrian refugees use antenatal vitamin supplementations at similar rates to Turkish citizens and obstetric and perinatal outcomes are similar between the groups.

**PRECIS:** We retrospectively analyzed the obstetric and perinatal outcomes and mode of delivery for Syrian refugees compared with Turkish citizens. Our data showed similar pregnancy outcomes among Syrian refugee women with Turkish women.

## Introduction

The war startedin Syria, resulted with immigration of Syrians to neighbouring countries such as Jordan, Lebanon, and Turkey. Millions of refugees entered Turkey, some refugees started to live in camps near the Syrian border but most were scattered around Turkey; some rented apartments, some started living with relatives or friends. Such a constrained movement naturally caused many severe health problems for the refugees but also negatively affected reproductive health and antenatal care for pregnant refugees^(1)^. İstanbul is one of the cities to which refugees immigrated intensely. According to the records of the Turkish Government Disaster and Emergency Management Agency, the number of Syrian refugees reached 2.7 million by March 2016, with approximetely 600.000 residing in İstanbul, thousands of whom were women of reproductive age^(2)^. Refugees at childbearing age and pregnant refugees face many difficulties, dealing with changing family dynamics, and assimilating into a new society while fearing for their safety^(3,4)^. They also have no medical insurance and consequently have problems reaching medical support in some countries. The Turkish government has provided free healthcare for Syrian refugees, so they can access medical treatment and support just like Turkish citizens without paying any money, including the drugs prescribed. Accordingly, there is no obstruction for Syrian refugees to obtain health care or medication in Turkey. Nevertheless, it is reported in the literature from different countries that the prevalence of poor reproductive health outcomes and antenatal complications such as preterm labour, low birthweight (LBW), increased incidence of cesarean sections (CS), bleeding during delivery, and increased puerperal infections are seen more often in refugee populations^(5,6,7)^. The aim of the current study was to compare the obstetric and perinatal outcomes of Syrian refugee women and their Turkish counterparts who gave birth at a tertiary center in İstanbul.

## Materials and Methods

A retrospective study between January 2016 and May 2017 at Süleymaniye Maternity Research and Training Hospital was planned. The birth records of 705 Syrian refugee and 750 Turkish pregnant women were retrospectively analyzed. One Syrian refugee and 6 Turkish women were excluded because the they had missing data. Finally, the records of 704 Syrian refugees and 744 Turkish pregnant women were included. The study was approved by the Süleymaniye Maternity Research and Training Hospital Local Ethics Committee (approval number: 2017/E 4737). Demographic data including maternal age, gravidy, complete blood count, presence of gestational diabetes mellitus (GDM) or preeclampsia were obtained. Smoking status and use of folic acid and iron supplementation during the patients’ pregnancies were noted. Gestational age at delivery, mode of delivery, the newborn’s 1^st^ and 5^th^ minute Appearance, Pulse, Grimace response, Activity, Respiration (APGAR) scores, birth weight, and if necessary, admission to neonatal intensive care unit (NICU) status was established. Complications developed during delivery, requirement of blood transfusion, and length of hospitalization of the mother were noted. All data was extracted from our hospital’s database system. Patients with systemic disorders such as pre-existing diabetes mellitus (type 1 or type 2), autoimmune disorders, acute or chronic active infections, heart diseases, and hematologic disorders were excluded from both groups. Gestational age was calculated according to the patients’ last menstrual period (LMP). For patients who did not know their LMP, gestational age was calculated according to the first trimester ultrasound of the patient from her file. 

### Statistical Analysis

Descriptive statistics are stated as percentage, mean ± standard deviation, median, frequency, ratio, minimum and and maximum. The Mann-Whitney U test was used for independent quantitative data. The chi-square test was used for independent qualitative data, if the chi-square test was not relevant, Fisher’s exact test was used. Logistic regression was performed to identify independent factors associated with the mode of cesarean delivery for the Turkish women and Syrian refugees. The distribution of variables was analyzed using the Kolmogorov-Smirnov test. The SPSS software program version 22.0 was used for statistical analyses. P<0.05 was considered statistically significant. 

## Results

The mean age for Syrian mothers was  23±4.3 years and 22.9±4.7 years for the Turkish mothers, and this was not statistically different. There was no difference between the groups in terms of gravidy and body mass index (BMI) results (Table 1). Although the rate of multiple pregnancies and hepatitis B surface antigen positivity was more frequent in the refugee population than in the Turkish subjects, this difference did not reach statistical significance. Hemoglobin and hematocrit values were also similar between the Syrian and Turkish patients (11.5±1.5 g/dL vs 11.6±1.6 g/dL; 33.1±7.3% vs 33.9±6.2%, respectively; p>0.05). Cigarette smoking was significantly higher among Turkish women than in the Syrians (14% vs 2%, respectively; p<0.05). The use of folic acid and iron supplementation during pregnancy was similar between the groups (Table 1). GDM was significantly higher for the Turkish population but preeclampsia rates were similar between the groups. 

Gestational age at delivery was significantly higher among Turkish women when compared with the Syrian refugees (37.7±2.3 vs 36.4±2.3 weeks, respectively; p<0.05), but there was no significant difference for the birtweights’ of the newborns (Table 2). The comparison of deliveries, neonatal outcomes, and obstetric complications is shown in Table 2. When the route of delivery was analyzed, cesarean rates were significantly higher for Turkish patients than for Syrian refugees (42.7% vs 32.7%, respectively; p<0.05). The main reason for CS was previous cesarean for both groups. The second most common indication for CS was nonprogressive labor for Turkish women (27.2%) and breech presentation for Syrian women (24.3%). Additionally, we perfomed logistic regression analysis for the parameters that increased cesarean delivery probability for both groups and found that increasing age, presence of preeclampsia, and multiple pregnancies increased cesarean delivery rates, and conversely, as BMI decreased, cesarean rates also decreasd (Table 3). Admission to the NICU and fetal anomaly rates for the newborn were similar between the groups and the most common symptom for NICU admission was respiratory distress. First and 5^th^ minute APGAR scores were also similar between both groups. Length of hospitalization was significantly shorter among Syrian refugees (1.6±0.8 vs 1.8±1.6 days, respectively; p<0.05). One of the main endpoints of this study was to compare the complications that developed during delivery, and although obstetric complications were seen more often in Syrian refugees, it did not reach statistical significance (8.1% vs 9.7%; p=0.28). The most common maternal complication was postpartum hemorrage and blood transfusion for both Syrian refugees and Turkish citizens (4.6% vs 4.4%, respectively; p>0.05). The second most common maternal complication was 3^rd^ degree perineal laceration for Turkish women (1.4%) and maternal infection for Syrian refugees (2%).

## Discussion

Syrian civil war caused displacement of millions of Syrians and the number of Syrian refugees giving birth in Turkey is increasing over time. Women in conflict areas may experience poorer pregnancy outcomes, including increased fetal mortality^(8)^, low birth weight^(9)^, premature labor, antenatal complications, and an increase in puerperal infections^(5)^ compared with pre-conflict levels. Several studies from Lebanon, Jordan, and Turkey reported higher cesarean rates for Syrian refugees than their own citizens, but different from them, we found higher cesarean rates for Turkish citizens^(1,7,10)^. Higher medico-legal anxiety related with Turkish pregnant women may be one of the reasons for such a condition. Similar to Alnuaimi et al.^(1)^ our data also supports that previous cesarean was the most common cause of another cesarean. The second most common indication for cesarean delivery was nonprogressive labor for Turkish women and breech presentation for Syrian women. Although the cesarean rate for breech presentation seems to be high, when it is corrected for all Syrian deliveries, the cesarean rate for breech presentation was 7.9% (56/704). Patients who are known to need cesarean for breech presentation are referred to our hospital from other government and private hospitals because we are a reference hospital. These data support that the most important point to decrease cesarean rates is to decrease primary CS rates. A study similar to ours was performed by Demirci et al.^(11) ^and they analyzed birth characteristics of Syrian refugees and Turkish citizens in Turkey in 2015. They reported lower hemoglobin values, lower length of hospitalization, and lower birthweight for the newborn of Syrian refugees compared with Turkish women. On the other hand, they reported higher cesarean delivery rates and higher GDM rates for Turkish citizens when compared with Syrian refugees. All of these findings were similar to our study, except for hemoglobin values. Our study demonstrated similar hemoglobin and hematocrit levels betwen Syrian refugees and Turkish citizens. Use of iron supplementation was similar between the groups in our study population, so this may account for the similar hemoglobin-hematocrit values. Kandasamy et al.^(3) ^reported similar GDM and preeclampsia rates between refugee women and control patients. In our study, the rate of GDM was significantly higher among Turkish citizens but preeclampsia rates were similar between the groups. Our clinical experience shows that there is higher compliance between Turkish citizens to glucose tolerance tests to diagnose GDM during antenatal period and this may cause the result of higher GDM diagnoses for Turkish pregnant women. On the other hand, preeclampsia is a diagnosis made by doctors to hospitalized patients according to their clinical and laboratory results. Reese Masterson et al.^(6) ^reported that the rate of preterm delivery, low birth weight, and bleeding during delivery occurred more frequently in Syrian refugee women when compared with control patients. Although Reese Masterson et al.^(6) ^ and Büyüktiryaki et al.^(7)^ reported higher rates of LBW neonates for Syrian refugees compared with Lebanese and Turkish citizens, the reults of Erenel et al.^(12) ^and our results do not support this finding because we found no difference for the newborns’ birthweights. Iron deficiency may increase the risk of LBW^(13)^ but our hemoglobin and hematocrit results showed no iron deficiency for refugees. Moreover, their iron supplementation was similar to the Turkish pregnant population so this may be a possible explanation for the normal-weight newborns. The APGAR scores of Syrian newborn babies were not lower than their Turkish counterparts and NICU admission rates were similar in our study; these data are also different from some other reports^(1,14)^. These similar APGAR scores and NICU admission rates may be because the newborns’ weights and congenital anomaly rates were similar between the groups in our study. The length of hospitalization was longer for Turkish citizens than Syrian refugees. This could be attributed to the higher rate of cesarean delivery because length of hospitalization is higher in cesarean deliveries. 

### Study Limitations

This study also has some limitations. First, it was a retropective study so the data that we could obtain was limited to what we could find in the records of the patients. It would be better if we could obtain much more data about the newborn’s NICU period. Furthermore, this study was performed at a tertiary center in İstanbul and the results may be different for refugees living in camps and for places where there limitations for refugees to reach health care providers. Previous studies of refugees from different countries identified the cost of health care and security concerns as barriers to women seeking health care but this is not valid for Turkey because our country provides health care to refugees without any limitation and free of charge. A government report noted that 90% of Syrian refugees in camps and 60% of those outside of camps used health services in Turkey and were satisfied with them^(15)^.

## Conclusion

In conlusion, as we started to collect the data, we expected that morbidity among Syrian refugee pregnant women and infants would be more common than Turkish citizens. However, according to our results, although there were some differences between the groups according to some headings, pregnancy outcomes among Syrian refugee women appears to be similar to those of Turkish citizens. The policy of the Turkish government about Syrian refugees may have a positive effect on these results, but an international contribution is essential to minimize the effect of the Syrian crisis both for Turkey and also for the sake of the refugees.

## Figures and Tables

**Table 1:**
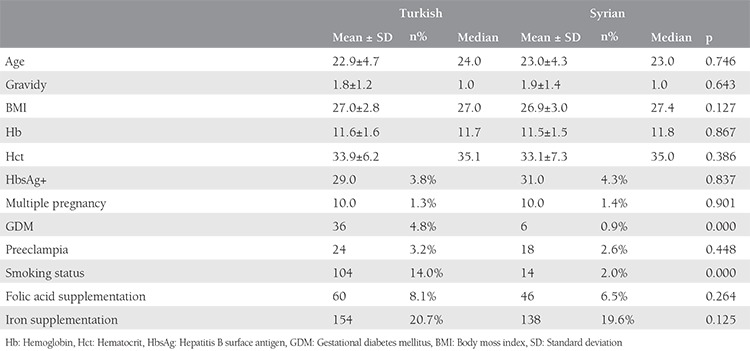
Demographic and clinic characteristics of both groups

**Table 2:**
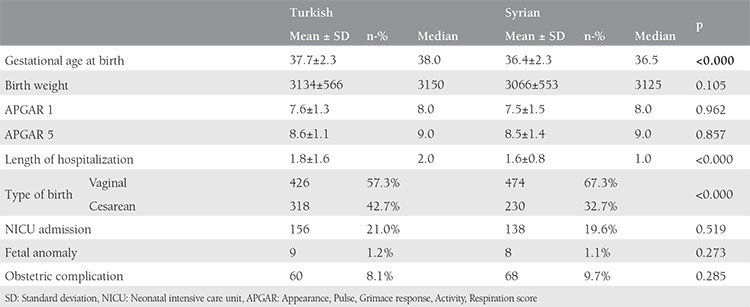
Comparison of obstetric and neonatal outcomes

**Table 3:**
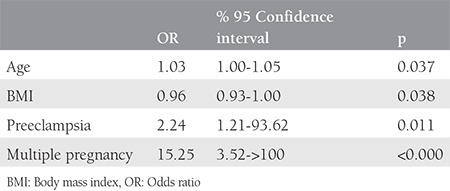
Factors independently associated with cesarean delivery including both groups
